# Mind the Gap: Sex-Specific Drivers of Human Papillomavirus Vaccination Uptake in Serbian University Students

**DOI:** 10.3390/ejihpe15090189

**Published:** 2025-09-19

**Authors:** Vida Jeremić Stojković, Stefan Mandić-Rajčević, Dejana Vuković, Mila Paunić, Snežana Stojanović Ristić, Marija Obradović, Smiljana Cvjetković

**Affiliations:** 1 Department of Humanities, Faculty of Medicine, University of Belgrade, 11000 Belgrade, Serbia; vida.jeremic-stojkovic@med.bg.ac.rs; 2 Institute of Social Medicine, Faculty of Medicine, University of Belgrade and School of Public Health and Health Management, Faculty of Medicine, University of Belgrade, 11000 Belgrade, Serbia; stefan.mandic-rajcevic@med.bg.ac.rs (S.M.-R.); dejana.vukovic@med.bg.ac.rs (D.V.); 3 Institute for Students’ Health of Belgrade, 11000 Belgrade, Serbia; mila.paunic@zzzzsbg.rs (M.P.); opsta.medicina@zzzzsbg.rs (S.S.R.); ginekologija@zzzzsbg.rs (M.O.)

**Keywords:** papillomavirus vaccines, vaccination acceptance, cross-sectional studies, university students, information seeking behavior

## Abstract

Despite proven effectiveness, human papillomavirus (HPV) vaccination uptake remains suboptimal in many countries. The aim of this study was to explore differences in beliefs about HPV and HPV vaccination, the information environment and social influences shaping vaccination decisions between male and female undergraduate university students in Belgrade, and to identify sex-specific factors associated with HPV vaccine uptake. A cross-sectional study was conducted between April and December 2024. An online questionnaire was completed by 1529 female and 423 male students who were either receiving their second or third dose of the nonavalent HPV vaccine, or accessing general healthcare services at the general medicine department of the Institute for Students’ Health of Belgrade. Hierarchical logistic regression was used to identify predictors of HPV vaccine uptake in male and female students. Among female students, HPV vaccine uptake was associated with stronger beliefs in vaccine efficacy (OR = 2.01, 95% CI: 1.50–2.69) and safety (OR = 2.29, 95% CI: 1.69–3.10), lower perceived lack of information (OR = 0.71, 95% CI: 0.60–0.84), and social influence of family members, (OR = 1.45, 95% CI: 1.04–2.03), colleagues (OR = 1.62, 95% CI: 1.01–2.59) and media (OR = 1.92, CI: 1.10–3.37). Among male students, vaccine uptake was associated with stronger beliefs in vaccine efficacy (OR = 2.14, 95% CI: 1.37–3.34), lower perceived lack of information (OR = 0.71, 95% CI: 0.52–0.98), more frequent reliance on scientific literature (OR = 1.50, 95% CI: 1.15–1.97) and family (OR = 1.37, 95% CI: 1.07–1.75) and less frequent use of YouTube (OR = 0.70, CI: 0.53–0.92) as sources of information, and social influence of family (OR = 1.83, 95% CI: 1.03–3.24). This study highlights significant sex differences in factors influencing HPV vaccine uptake, indicating that tailored approach is required in designing vaccine promotion strategies. Strengthening communication on efficacy and safety, improving access to reliable information, and addressing sex-specific concerns such as safety and financial barriers in females and misinformation in males could improve uptake and equitable HPV protection.

## 1. Introduction

Cervical cancer ranks as the fifth leading cause of cancer among women in Serbia, and the second most common cancer in women aged 15 to 44 years, with the incidence being significantly higher compared to Southern Europe (18.7 vs. 7.72 per 100,000, respectively) (Bruni et al., 2023b). Similarly, cervical cancer mortality in Serbia is higher compared to Southern Europe (14.2 vs. 4.72 per 100,000, respectively). Although organized cervical cancer screening has been introduced in 2012, the current screening coverage for cervical cancer on the territory of Serbia varies between 35% and 68% (Djordjevic et al., 2024). Therefore, it can be concluded that the issue of preventing cervical cancer presents a pressing public health challenge in Serbia.

At this moment, human papillomavirus (HPV) vaccine is the only recommended effective way of preventing cancers caused by persistent HPV infection. HPV vaccine is routinely recommended to girls as early as 9 years, with the recommendation being amended to include young adults until the age of 26 (Meites, 2019). Extensive research has shown that HPV vaccines demonstrated an acceptable safety profile, with no consistent evidence of an increased risk of serious adverse events (Phillips et al., 2018). Improving HPV vaccination rates is critical given that HPV vaccination could prevent 90% of HPV-related cancers (Chesson et al., 2019). Research indicates that in some high-income countries, vaccination rates can exceed 75%, but many other countries continue to experience low uptake (Altobelli et al., 2019). Barriers to HPV vaccination are complex and multifactorial, including sociocultural, economic and political factors (Cangelosi et al., 2025). One of the major obstacles to HPV vaccination is the limited awareness and understanding of its importance, particularly in low- and middle-income countries (LMICs) (Cangelosi et al., 2025). In addition, doubts about vaccine effectiveness, beliefs that vaccination is not necessary, and worries about safety and potential side effects represent further barriers to the successful implementation of HPV vaccination (Urrutia et al., 2023). To ensure high levels of acceptance, interventions in HPV vaccination must rely on comprehensive understanding of the barriers to HPV vaccination in local contexts, community needs and cultural specificities, in order to build trust and increase awareness of HPV and vaccine importance (Cangelosi et al., 2025).

Research from multiple countries in previous decades has demonstrated that expanding HPV vaccination programs to include males is a highly cost-effective preventive strategy, decreasing incidence of anogenital cancers, certain head and neck cancers, precancerous lesions, and genital warts in both men and women (Elbasha & Dasbach, 2010; Burger et al., 2014; Cheng et al., 2020). An important rationale for vaccinating males against HPV is their role in transmitting the virus to female partners, often unknowingly, as HPV infection in men is typically asymptomatic (Naidoo et al., 2024). A systematic review published in 2023 reported that approximately one-third of men aged 15 years and older were infected with at least one type of human papillomavirus (HPV), and that around one-fifth harbored one or more high-risk or oncogenic HPV types (Bruni et al., 2023a). Achieving high vaccine coverage across both genders would support the development of herd immunity and significantly reduce the overall burden of HPV.

In Serbia, recommended immunization against diseases caused by human papillomaviruses for both girls and boys aged 9–19 years, funded by the Health Insurance Fund of Serbia, began in June 2022, with the use of the freely available nonavalent HPV vaccine (Gardasil 9). Although HPV vaccination is most effective when administered at younger ages, prior to sexual activity, vaccination in young adulthood still provides partial protective benefits (Ellingson et al., 2023). Based on this rationale, in April 2024, the Fund provided free doses for undergraduate male and female students age 19–26 through the Institute for Students’ Health of Belgrade. Currently, no data exist about HPV vaccination coverage in the undergraduate student population, and general HPV vaccination coverage varies greatly between districts of Serbia, ranging from 4% to 12%.

University students present a distinctive population of young adults who autonomously make health-related decisions, including vaccination decision. Previous studies conducted among young adults suggest that confidence in HPV vaccine efficacy and safety, as well as perception of risks and severity associated with HPV-caused diseases play significant role in their decision-making (D’Errico et al., 2020). Confidence in vaccines and complacency (perceived lack of need for vaccinations, where the risks of vaccine-preventable disease are perceived as low) are widely recognized as key components of the global phenomenon of vaccine hesitancy (MacDonald, 2015). Information environment, including informational processes and sources of information can also affect the decision-making process regarding vaccination. For example, previous studies on routine immunization show that individuals who delay or refuse vaccination are more likely to use internet as their main source of information about vaccines (Smith et al., 2017). Social influence can also present potentially critical factor of HPV vaccine uptake, since previous studies suggest that lack of perceived social approval of vaccination from social networks, family members, community members, organized groups, institutions or media presents significant barrier to vaccination uptake (Kaufman et al., 2021). Although university students do not present the primary target for HPV vaccination, we were motivated to conduct the study in this population following the Fund’s decision to provide free vaccination for students, which resulted in a large response. Moreover, understanding the beliefs and perspectives of university students is important, as they may serve as advocates for younger groups. In addition, university students can play a strategic role as multipliers of information within their community.

According to the Behavioral Drivers Model, which presents a transtheoretical, comprehensive model of determinants of behavior, there are three levels of drivers, each contributing to the outcome behavior: psychological (individual cognitive and emotional factors), sociological (determinants related to interactions within families, communities, groups and society at large) and environmental (institutions, policies, systems and services, infrastructures, information, etc.) (Petit, 2019). For the purpose of this study, we decided to investigate selected factors at all three levels, with the hypothesis that HPV vaccine-related beliefs (psychological level), social influencers (sociological level) and information environment (environmental level) would significantly predict HPV vaccine uptake.

Initially, HPV vaccine testing and marketing to prevent cervical cancer have been specifically focused on females, which led to gender biases in the perception of the need for vaccination (Daley et al., 2016). The strong association between HPV and cervical cancer has drawn attention away from the need to prevent other HPV-related cancers, such as oropharyngeal cancers, which impact both men and women. As HPV vaccine has been approved for males with years of delay, females usually manifest higher awareness, knowledge and interest for HPV vaccination (Dodd et al., 2014; McBride & Singh, 2018). Several studies indicate that different factors might affect HPV vaccination decision-making in males and females (Theotonio dos Santos et al., 2025; Brunelli et al., 2021; Chen et al., 2021), implying the need to explore these differences and their impact on vaccine uptake in both sexes. Therefore, the primary aim of this study was to examine differences between male and female undergraduate university students’ beliefs regarding HPV and HPV vaccination, their information environment and the main agents of social influence affecting vaccination decision. The secondary aim was to identify factors associated with HPV vaccine uptake among male and female students.

## 2. Materials and Methods

### 2.1. Study Design and Setting

This was a cross-sectional study, conducted in the period June-December 2024 on a convenience sample of undergraduate university students at The Institute for Students’ Health of Belgrade (Students’ Policlinic). The primary role of the Students’ Policlinic is to provide programs of preventive care and treatment to student population, customized to focus on most prevalent health problems of students, such as problems related to reproductive health, eating disorders, mental health, skin and teeth. Data were collected at the general medicine department, where students routinely receive general healthcare services, and where vaccination with the second and third dose of the nonavalent HPV vaccine was organized during the study period.

### 2.2. Participants

Eligible participants were undergraduate university students (aged 18–27 years) attending the Students’ Policlinic for either general healthcare services or for HPV vaccination appointment. Students were recruited by members of the research team while waiting for their appointment. Research team members were organized to cover the morning shifts and most of the afternoon shifts at the Students’ Policlinic (from 8.00 a.m. to 4:00 p.m.) every working day during the study period, approaching every student attending clinic during these hours. Approached students were verbally informed and provided information sheets about the study aims, methodology and participants’ right. If they accepted to participate, they were given a QR code, which referred to the Research Electronic Data Capture (REDCap) survey hosted on secure servers of the Faculty of Medicine, University of Belgrade. Data were collected by a self-administered questionnaire.

### 2.3. Study Instrument and Variables

The questionnaire was developed based on the comprehensive literature review on drivers of vaccination behavior (Larson et al., 2014; Kaufman et al., 2021; Díaz Crescitelli et al., 2020; Karafillakis et al., 2019). The questionnaire was pilot tested in a group of university students (*N* = 20) before the beginning of this study, at the same place where the actual data collection would take place. Readability and understandability of all items were confirmed. The questionnaire included following sections:

1. **HPV vaccination status** was measured by a single item assessing whether the participant took the HPV vaccine, with the binary (YES/NO) response;
2. **Sociodemographic characteristics** included sex, age, faculty group, religiousness, self-assessed financial status, and relationship status. Religiousness was originally measured as a continuous numerical variable ranging from 1, indicating “not religious at all”, to 100, indicating “extremely religious”. For analytical and interpretative purposes it was divided into quintiles, creating conceptually meaningful categories: 0–20 completely nonreligious, 20.1–40 moderately nonreligious, 40.1–60 neither nonreligious nor religious, 60.1–80 moderately religious, and 80.1–100 completely religious. This categorization facilitates clearer communication of results and comparison across groups. Self-assessed financial status was measured on a five-point scale(very good/good/average/bad/very bad). We used a self-assessed measure of financial status to reflect students’ perceived economic position, which is particularly relevant for behaviors such as vaccination uptake. Categorizing responses into five levels allows for clear interpretation and meaningful comparisons across subgroups. Relationship status was categorized as in a long-term relationship (including marriage), single (including divorced) and other/it’s complicated).
3. **HPV and HPV vaccine-related beliefs** included three short scales on a five-point agreement Likert scales (ranging from 1 “strongly disagree” to 5 “strongly agree”). The total score for each scale was calculated by summing the responses to all items, and dividing that sum with the number of items. Items with negative connotation were reversely coded when calculating the total scores, ensuring that all items are aligned in the same direction, so that higher scores consistently reflect a higher intensity of the construct being measured. The total score range for each scale was divided in four quartiles: 1–1.99 (highly negative), 2–2.99 (moderately negative), 3–3.99 (moderately positive) and 4–5 (highly positive).
  - Perceived vaccine efficacy: three items, α = 0.85;
  - Perceived vaccine safety: five items, α = 0.84 (items 2 and 5 were reversely coded);
  - Perceived danger of HPV vaccine-preventable diseases: four items, α = 0.79 (items 1, 2, 3 and 4 were reversely coded).
4. **Information environment** included
  - Perceived lack of information was evaluated with four questions on a five-point Likert scale ranging from 1 “strongly disagree” to 5 “strongly agree” (α = 0.89). Item 4 was reversely coded. Higher score indicated stronger feeling of the lack of information.
  - Use of information sources was evaluated by twelve items inquiring about the frequency of use of selected sources of information regarding HPV and HPV vaccines on five-point Likert scale ranging from 1 “Never” to 5 “Regularly”.
5. **Social influence** was assessed by asking participants to select three of the listed social influencers (family, friends, peers, colleagues from the faculty, community members, national health authorities, religious leaders, healthcare providers, the government, the media) that have the strongest influence on their vaccination decision.

### 2.4. Bias

Several steps were taken to reduce potential sources of bias in this study. Selection bias may have occurred because not all eligible students could be approached. Information bias was addressed by using pilot-tested instruments. Furthermore, assuring anonymity and confidentiality were measures taken to reduce social desirability bias. Recall bias regarding vaccination status was likely minimal, as many participants were attending the clinic to receive the vaccine.

### 2.5. Statistical Analysis

Descriptive statistics were used to characterize the sample and study variables. Categorical variables were presented as frequencies and percentages, while continuous variables were presented as means ± standard deviation. The differences between male and female participants with regard to sociodemographic characteristics, vaccine beliefs, perceived lack of information, use of information sources and social influence were analyzed using Chi-square, Fisher’s exact test, the t-test and the Mann–Whitney U test.

Factors associated with the HPV vaccination status as the main outcome variable were analyzed separately for males and females using hierarchical multivariate logistic regression. Variables were first screened in univariate analyses, and those showing associations with vaccination status at *p* < 0.05 were considered for inclusion in the multivariate models.

Hierarchical approach involves adding block of variables sequentially into the regression model, to assess their incremental predictive power. Blocks of predictors were added based on theoretical and empirical considerations. Sociodemographic characteristics were entered first as basic background factors. Beliefs regarding HPV vaccination were added in the second block, reflecting individual cognitive and emotional drivers. Variables related to information environment were entered in the third block, to capture the influence of exposure to information. Finally, social influence variables were entered in the fourth block, to assess contextual and relational effects. To include categorical variables with multiple values in the regression models, dummy variables were created.

Nagelkerke R Square was employed as a measure of the goodness of fit for hierarchical multivariate regression models. Multicollinearity was assessed by examining tolerance and variance inflation factor (VIF) values.

All *p* values less than 0.05 were considered significant. Data were analyzed using SPSS 20.0 (IBM Corp. Released 2011. IBM SPSS Statistics for Windows, Version 20.0. Armonk, NY, USA: IBM Corp.).

### 2.6. Ethical Consideration

This study was approved by the Ethics Committee of the Faculty of Medicine (Decision No. 25/VI-6) and the Ethics Committee of the Institute for Students’ Health of Belgrade (Decision No. 1321/2). This study was voluntary and anonymous. No personal identifying data were collected and no incentives provided. Participants were informed about this study verbally and through information sheets, and they provided their consent by ticking the specified box at the beginning of the electronic survey. The consent was a condition to continue with filling the electronic data form.

This study was reported in accordance with the Strengthening the Reporting of Observational Studies in Epidemiology (STROBE) Statement for cross-sectional studies (Von Elm et al., 2007). The completed checklist is provided in the Supplementary Material.

## 3. Results

The total of 1952 students completed the questionnaire. Sociodemographic characteristics of the sample are presented in Table 1. Majority were females (78.3%,), with the mean age of 21.8 years. While there were no differences between male and female participants with respect to the faculty of study, male students more often rated their financial situation as very good or good, and were more likely to describe themselves as completely nonreligious and single. Female students more often reported moderate financial status, identified as neither nonreligious nor religious, and were more often in long-term relationships.

With respect to vaccination status, just over half of the sample had received the HPV vaccine, with uptake notably higher among women than men (*p* < 0.001).

Participants in our sample reported very positive beliefs about HPV vaccine efficacy (Table 2), with mean scores indicating high confidence overall, particularly among female students. Vaccinated students demonstrated significantly greater confidence in vaccine efficacy compared to their unvaccinated peers, in both male and female subgroups.

The majority of participating students considered the HPV vaccine important for their health, a view more strongly endorsed by women than men, and by vaccinated students across both sexes. Also, vast majority agreed that HPV vaccine can prevent HPV infection and protects from oncogenic strains of HPV virus, with female students again showing greater endorsement of these beliefs compared to their male peers. Vaccinated students in both male and female subgroups were more likely to share these beliefs than their unvaccinated counterparts.

Participating students’ beliefs regarding HPV vaccine safety were generally positive across the sample (Table 3). Average scores were significantly higher among female students, and among vaccinated students of both sexes. Compared to their counterparts, female students and vaccinated students were more likely to reject the claim that HPV vaccine can cause infertility, more likely to believe that HPV vaccines are safe, and more often endorsed the view that serious adverse reactions are extremely rare They were also more likely to reject the idea that HPV vaccines have not been adequately studied to confirm their safety.

Beliefs regarding the danger of HPV vaccine preventable diseases differed markedly between male and female respondents, and between vaccinated and non-vaccinated of both sexes (Table 4). Average scores were significantly higher among female students and among vaccinated students across both sexes. Male students and unvaccinated participants of both sexes were more likely to share beliefs that HPV infection is not so common so they are at low risk of contracting it, and that HPV infection can be prevented by other means Also, they were more likely to believe that disease caused by HPV are not that common, and that HPV infection is not dangerous since it resolves spontaneously.

Surveyed students most frequently relied on internet portals, social networks, friends, chosen physician and family as sources of vaccine-related information (Table 5). Female students reported more frequent use of nearly all selected information sources compared with males, particularly scientific literature, and traditional media such as national and regional TV channels, but also electronic media such as internet portals, YouTube channels and social networks, and interpersonal (family, friends) and professional sources (chosen physician, health professionals in media). Vaccinated students of both sexes reported more frequent reliance on scientific literature, internet portals, social networks, family, friends, chosen physician and healthcare professionals in media. Among males, vaccinated students more often cited national TV channels and religious leaders as sources compared to unvaccinated males, while among females, unvaccinated students more often relied on religious leaders and governmental sources of information compared to their vaccinated peers.

Perceived lack of information about HPV vaccination was generally low among participants, with no significant differences in average scores between male and female respondents. However, unvaccinated students of both sexes reported higher scores compared to their vaccinated peers (Table 6). Approximately 20% of participants reported having difficulties when deciding whether to get vaccinated against HPV since there is a lack of information, and reported feeling confused due to incomplete or contradictory information regarding HPV vaccines they come across, with the unvaccinated students of both sexes being more likely to report these issues. The largest proportion of respondents indicated that they have all information regarding HPV vaccination they need, with vaccinated students of both sexes more frequently endorsing this view.

Most of the surveyed students identified family members, healthcare workers and friends as having the strongest social influence on their intention to get vaccinated against HPV (Table 7). Female students were more influenced by family members and healthcare workers, whereas male students reported greater influence by peers, national health authorities and government. Vaccinated students of both sexes were significantly more likely than unvaccinated peers to report influence from faculty colleagues. Among females, vaccinated students reported greater influence from friends and media, and lower influence from religious leaders compared to unvaccinated students.

Results of the hierarchical logistic regression analysis are presented in Table 8. Collinearity diagnostics indicated no problematic multicollinearity (all VIF < 5, tolerance > 0.1).

In Model 1, which included sociodemographic characteristics, 8.8% of the variance in vaccination status was explained. Older age increased the likelihood of vaccination (OR = 1.11, 95% CI: 1.05–1.18, *p* < 0.001). Compared to completely religious students, those identifying as completely nonreligious (OR = 2.69, 95% CI: 1.97–3.68, *p* < 0.001), moderately nonreligious (OR = 2.08, 95% CI: 1.40–3.07, *p* < 0.001), neither nonreligious nor religious (OR = 1.93, 95% CI: 1.39–2.66, *p* < 0.001), and moderately religious (OR = 1.62, 95% CI: 1.23–2.13, *p* < 0.01) were more likely to be vaccinated. Students from technology and engineering (OR = 0.45, 95% CI: 0.37–0.61, *p* < 0.001), science and mathematics (OR = 0.47, 95% CI: 0.32–0.70, *p* < 0.001), and social sciences and humanities (OR = 0.53, 95% CI: 0.41–0.69, *p* < 0.001) were less likely to be vaccinated compared to medical sciences.

In Model 2 (adding HPV vaccination beliefs), explained variance increased to 35.6%. Religiosity remained significant but attenuated, with only students identifying as neither nonreligious nor religious more likely to be vaccinated (OR = 1.50, 95% CI: 1.03–2.19, *p* < 0.05). Stronger beliefs in vaccine efficacy (OR = 2.26, 95% CI: 1.80–2.85, *p* < 0.001), safety (OR = 2.36, 95% CI: 1.89–2.95, *p* < 0.001), and greater perceived danger of HPV-related diseases (OR = 1.18, 95% CI: 1.01–1.38, *p* < 0.05) predicted vaccination.

In Model 3 (adding factors of information environment), explanatory power rose to 39.0%. Greater confidence in efficacy (OR = 1.97, 95% CI: 1.56–2.49, *p* < 0.001) and safety (OR = 1.83, 95% CI: 1.42–2.35, *p* < 0.001), lower perceived lack of information (OR = 0.74, 95% CI: 0.64–0.85, *p* < 0.001), and more frequent reliance on family (OR = 1.20, 95% CI: 1.07–1.35, *p* < 0.01) and chosen physician (OR = 1.12, 95% CI: 1.00–1.26, *p* < 0.05) as sources of information were associated with higher odds of vaccination.

In Model 4 (adding factors of social influence), explained variance increased to 40.3%. Vaccination was more likely among students with greater confidence in vaccine efficacy (OR = 1.98, 95% CI: 1.56–2.52, *p* < 0.001) and safety (OR = 1.80, 95% CI: 1.40–2.32, *p* < 0.001), lower perceived lack of information (OR = 0.73, 95% CI: 0.63–0.85, *p* < 0.001), more frequent reliance on chosen physicians (OR = 1.18, 95% CI: 1.05–1.32, *p* < 0.01) and less frequent use of YouTube (OR = 0.88, 95% CI: 0.77–0.99, *p* < 0.05). Strong social influence from family (OR = 1.64, 95% CI: 1.23–2.18, *p* < 0.01), colleagues (OR = 1.63, 95% CI: 1.09–2.44, *p* < 0.05), and media (OR = 1.81, 95% CI: 1.16–2.82, *p* < 0.05) also predicted uptake. Poor financial status emerged as a negative predictor (OR = 0.22, 95% CI: 0.07–0.71, *p* < 0.05).

Table 9 presents the hierarchical multiple logistic regression analysis of variables significantly associated with vaccination status in male respondents. Collinearity diagnostics showed no evidence of multicollinearity among the predictors (tolerance > 0.1; VIF < 5). In the first model which included sociodemographic variables, studying technology and engineering sciences (OR = 0.50, 95% CI: 0.27–0.92, *p* < 0.05) and social sciences and humanities (OR = 0.32, 95% CI: 0.17–0.60, *p* < 0.001) was significantly associated with the lower odds of being vaccinated against HPV. The Nagelkerke R^2^ for this model was 0.074, indicating a small proportion of variance being explained by sociodemographic factors.

Including beliefs about HPV vaccination in the second model, revealed that having more positive beliefs regarding HPV vaccine efficacy significantly increased the likelihood of vaccination (OR = 2.48, 95% CI: 1.60–3.83, *p* < 0.001),improving the explanatory power of the model (Nagelkerke R^2^ = 0.228).

When factors of information environment (perceived lack of information and frequency of use of selected information sources) were included in the third model, more frequent reliance on scientific literature (OR = 1.45, 95% CI: 1.12–1.89, *p* < 0.01) and family (OR = 1.45, 95% CI: 1.15–1.84, *p* < 0.01) as sources and less frequent use of YouTube channels as a source (OR = 0.72, 95% CI: 0.55–0.94, *p* < 0.05) were associated with higher chances of being vaccinated against HPV, with more positive beliefs regarding vaccine efficacy remaining as significant predictor (OR = 2.21, 95% CI: 1.42–3.44, *p* < 0.001). The predictive power of the model was further improved (Nagelkerke R^2^ = 0.313).

The final fourth model, which included socioeconomic characteristics, vaccine beliefs, factors of information environment and factors of social influence, explained 33.1% of variance in the vaccination. Key factors associated with higher vaccination likelihood in male students included stronger beliefs in vaccine efficacy (OR = 2.14, 95% CI: 1.37–3.34, *p* < 0.05, lower perceived lack of information (OR = 0.71, 95% CI: 0.52–0.98, *p* < 0.05), greater reliance on scientific literature (OR = 1.50, 95% CI: 1.15–1.97, *p* < 0.05) and family (OR = 1.37, 95% CI: 1.07–1.75, *p* < 0.05),less frequent use of YouTube (OR = 0.70, 95% CI: 0.53–0.92, *p* < 0.05) as sources of information, and identifying family as the strongest social influence on the vaccination decision (OR = 1.83, 95% CI: 1.03–3.24, *p* < 0.001)

Results of the hierarchical multiple logistic regression analysis of variables significantly associated with vaccination status in female respondents are presented in Table 10. Collinearity checks confirmed that the independent variables did not exhibit problematic multicollinearity (all VIF < 5, tolerance > 0.1). In the first model, which included sociodemographic characteristics, only 11.2% of variance in vaccination status was explained. Older age increased the likelihood of vaccination (OR = 1.16, 95% CI: 1.08–1.24, *p* < 0.001). Compared to completely religious students, those who identified as completely nonreligious (OR = 3.74, 95% CI: 2.54–5.52, *p* < 0.001), moderately nonreligious (OR = 2.47, 95% CI: 1.55–3.93, *p* < 0.001), neither nonreligious nor religious (OR = 2.10, 95% CI: 1.46–3.02, *p* < 0.001), and moderately religious (OR = 1.88, 95% CI: 1.37–2.57, *p* < 0.001) were more likely to be vaccinated. Students from technology and engineering (OR = 0.50, 95% CI: 0.35–0.72, *p* < 0.001), science and mathematics (OR = 0.44, 95% CI: 0.28–0.69, *p* < 0.001), and social sciences and humanities (OR = 0.60, 95% CI: 0.45–0.81, *p* < 0.001) fields were less likely to be vaccinated compared to those in medical sciences.

In the second model, adding HPV vaccination beliefs in the second model substantially improved the predicting power (Nagelkerke R^2^ = 0.407). Religiosity remained significant predictor of vaccination status, though the effect sizes were smaller: completely nonreligious (OR = 1.66, 95% CI: 1.06–2.60, *p* < 0.05), neither nonreligious nor religious (OR = 1.65, 95% CI: 1.07–2.54, *p* < 0.05) and moderately religious (OR = 1.57, 95% CI: 1.08–2.28, *p* < 0.05) students were more likely to be vaccinated. Students reporting poor financial status had lower chances to be vaccinated (OR = 0.30, 95% CI: 0.09–0.94, *p* < 0.05). More positive beliefs about vaccine efficacy (OR = 2.18, 95% CI: 1.64–2.89, *p* < 0.001), and safety (OR = 3.14, 95% CI: 2.40–4.13, *p* < 0.001) were associated with higher chances of being vaccinated.

The third model, which included factors of information environment, further improving predictive power of the model (Nagelkerke R^2^ = 0.429). Moderately religious female students (OR = 1.57, 95% CI: 1.07–2.30, *p* < 0.05), with stronger confidence in vaccine efficacy (OR = 1.96, 95% CI: 1.48–2.62, *p* < 0.001) and safety (OR = 2.35, 95% CI: 1.74–3.18, *p* < 0.001), and lower perceived lack of information (OR = 0.71, 95% CI: 0.60–0.84, *p* < 0.001) were more likely to be vaccinated against HPV. Students reporting poor financial status (OR = 0.23, 95% CI: 0.07–0.75, *p* < 0.05) and frequent reliance on religious leaders as a source of information (OR = 0.80, 95% CI: 0.65–0.98, *p* < 0.05) had lower chances to be vaccinated.

In the fourth model, which included factors of social influence, 44% of variance in vaccination status was explained. Female respondents with stronger confidence in HPV vaccine efficacy (OR = 2.01, 95% CI: 1.50–2.69, *p* < 0.001) and safety (OR = 2.29, 95% CI: 1.69–3.10, *p* < 0.001), lower perceived lack of information (OR = 0.71, 95% CI: 0.60–0.84, *p* < 0.001), and who identified family members (OR = 1.45, 95% CI: 1.04–2.03, *p* < 0.05), colleagues (OR = 1.62, 95% CI: 1.01–2.59, *p* < 0.05) and media (OR = 1.92, 95% CI: 1.10–3.37), *p* < 0.05) as strongest social influencers had higher odds to be vaccinated against HPV. Poor financial status remained a negative predictor of vaccination status (OR = 0.22, 95% CI: 0.07–0.71, *p* < 0.05).

## 4. Discussion

### 4.1. Sex Differences in HPV Vaccine-Related Beliefs

Overall, most participating students had highly positive beliefs about HPV vaccine efficacy and safety, but male students were less confident. This pattern is consistent with results of several international studies (Aldawood et al., 2023; Dai et al., 2022), though not universal (Goldfarb & Comber, 2022). Vaccinated students of both sexes were more confident in HPV vaccine efficacy and safety, and perceived the risk of HPV as higher compared to unvaccinated peers, suggesting that both trust in the vaccine and risk perception are important drivers of vaccine uptake.

Low perceived HPV-related risk and susceptibility in male students have been observed in numerous studies conducted in previous decades, (Fontenot et al., 2014; Balcezak et al., 2022; Olusanya et al., 2023), and can be partially attributed to the fact that most HPV infections remain asymptomatic in both males and females (Bruni et al., 2023a), while there is a lack of routine screening approach in males Another explanation for the perception of low risk and susceptibility to HPV infection and related diseases, but also lower confidence in HPV vaccine efficacy and safety in males can lie in gendered promotional strategies targeting primarily female population, diminishing the perceived importance of receiving the vaccine in male population. Vaccine-promotion campaigns targeting male population and parents of boys should be better tailored to address male-specific concerns, emphasizing how HPV can affect men and the importance of vaccination.

### 4.2. Sex Differences in Factors of Information Environment

Approximately one-half of respondents reported having all the information regarding HPV vaccination they need, with no difference in the overall perception of lack of information between male and female students. However, unvaccinated students of both sexes reported a greater perceived lack of information, underscoring the critical role of adequate and accessible information in facilitating HPV vaccine uptake. Responding students most frequently relied on informal sources of HPV vaccine-related information (internet portals, social networks, friends and family), but women reported higher engagement with nearly all sources, which can be explained by generally higher interest of females for the issue of HPV vaccination.

It is generally posited that traditional media, such as television and radio, have a more substantial positive impact on vaccine uptake compared to newer forms of media, particularly social media (Odone et al., 2015; Catalan-Matamoros & Peñafiel-Saiz, 2019). This assumption is grounded in the view that traditional media are more effective in disseminating accurate and reliable information about vaccines to a broad audience, whereas social media platforms are more vulnerable to the spread of misinformation. However, the finding that majority of surveyed students of both sexes actually rely on internet portals and social networks as vaccine-related information sources suggests the need to reconstruct traditional understanding of health communication and reach out to target populations through sources they use most often. Vaccination campaigns targeting young adults should be developed to reach those who might not regularly receive vaccine information in the traditional media, employing information sources such as social media and digital channels to share accurate vaccine information.

### 4.3. Sex Differences in Factors of Social Influence

Family, healthcare workers, and friends were the strongest social influencers overall, with females more often guided by family and healthcare providers, while males were more receptive to the influence of government and health authorities. While family and healthcare setting are traditionally considered as being effective in driving healthcare choices, peer influence also entails great impact, which was established in various studies (Khurana et al., 2015; Verelst et al., 2019; Cocchio et al., 2020). The greater susceptibility of male students to the influence of state agents (national health authorities and government) underscores the strategic importance of national health authorities and governmental institutions as key messengers in vaccine promotion efforts targeting this population. The finding that vaccinated students of both sexes reported greater influence by their faculty colleagues compared to their unvaccinated peers suggests that peer networks within the academic environment may play an important role in shaping vaccination decisions. Faculty colleagues may serve as trusted sources of information and role models, implying the need to promote positive social norms in the professional environment

### 4.4. Predictors of HPV Vaccine Acceptance in Male and Female Students

Confidence in vaccine efficacy, perceived lack of information, and family influence emerged as common predictors of HPV vaccine acceptance across sexes, while vaccine safety concerns, financial status and colleagues/media influence were more salient among females, and reliance on scientific literature, family and YouTube as information sources among males.

These results are consistent with previous studies, which have identified perceived benefits, safety concerns, and inadequate information as central predictors of HPV vaccine uptake among young adults (Maier et al., 2015; Liu et al., 2020; Chen et al., 2021), as well as the importance of social influence from parents, family, and peers (LaJoie et al., 2018; Quinn & Lewin, 2019; Stout et al., 2020; Goldfarb & Comber, 2022). Taken together, these findings suggest that while confidence in efficacy, clear information, and family support are universal drivers of HPV vaccination, sex-specific differences should inform tailored interventions. Addressing vaccine safety concerns and financial barriers may be particularly important for female students, while promoting access to credible scientific information and countering misinformation on platforms such as YouTube may be more relevant for males. The consistent impact of family influence across sexes further emphasizes the need for family-centered communication strategies to increase vaccine acceptance.

### 4.5. Practical Implications

Evidence suggests that community-based interventions could be a key strategy in overcoming barriers to HPV vaccination acceptance (Brandt et al., 2019). However, for these efforts to be effective, they must respond to specific needs in local contexts—in our case, students’ concerns about HPV vaccine efficacy and safety, the strong influence of family, perceived lack of information and reliance on specific sources of information. Robust communication strategies are therefore not only essential to maintaining high and sustained HPV vaccine coverage (WHO, 2017), but also for tailoring vaccine promotion interventions to the realities of young adults’ decision-making in Serbia.

The finding that beliefs regarding HPV vaccine safety and efficacy were among the most influencing factors of students’ vaccine uptake points to the need to create interventions based on risk communication—vaccine mechanisms and efficacy, vaccine safety surveillance mechanisms and both common and expected and rare but serious vaccine side effects (Lewandowsky et al., 2023). As the research results suggest that vaccine safety beliefs were particularly determining in female students, while male students generally manifested lower perceived risk and susceptibility to HPV infection, interventions should be tailored to address these sex differences.

To address the issue of lack of information, adequate access to trustworthy information should be enabled, using credible spokespeople (primarily chosen physicians, as they were among the most frequently relied sources and most influential social agents among both sexes), using both formal and informal communication channels. Scientific results must be communicated clearly, transparently and consistently to avoid misinterpretation.

Since families strongly influence decisions about HPV vaccination in students and are often a key source of health information, it is essential that HPV vaccination promotion strategies actively involve and support family engagement. Considering the interpersonal trust and shared experiences that characterize family relationships (Frew et al., 2014; Hesse & Rauscher, 2016), vaccine promotion messaging and intervention that target families are likely to be more effective in promoting vaccination acceptance and adherence, as families are most likely to influence future vaccination decisions. Finally, the positive influence of university colleagues may be leveraged through student-led initiatives or peer education, complementing family-based and physician-led approaches, reinforcing positive attitudes toward HPV vaccination within the student community.

### 4.6. Limitations

Our study had several notable limitations. First, cross-sectional design of this study does not allow for identifying causal relationship between factors and HPV vaccine uptake. Although our sample included students attending the biggest university in Serbia, which enrolls students from all over the country, we cannot generalize results to other Serbian universities or non-student young adult populations, although our results can give guidance on how health behavior of these populations could be studied and improved. Since we recruited participants among students attending the clinic either for vaccination appointment or for other services, we cannot exclude selection bias, since students who attend the clinic may be more health-conscious, or already more engaged with preventive services compared to those who do not seek care. Lastly, although the anonymity and confidentiality were guaranteed, we cannot rule out the possibility of social desirability bias. An important strength of this sample is the high reliability of students’ self-reported vaccination status, since participants were attending the Policlinic specifically to receive their second or third dose of the HPV vaccine.

## 5. Conclusions

Our study revealed notable differences between male and female students in beliefs about HPV vaccination, the use of information sources related to the HPV vaccine, and the social influences shaping vaccination decisions. Male students exhibited lower confidence in HPV vaccine efficacy and safety, as well as lower perceived risk and susceptibility to HPV infection and HPV-related diseases, whereas female students reported more frequent use of nearly all selected sources of information, and acknowledged larger social influence of family members and healthcare workers. Furthermore, the factors associated with vaccine acceptance also varied between male and female students. While HPV vaccine uptake in both sexes was strongly predicted by confidence in vaccine efficacy, perceived lack of information and family influence, additional predictors differed—male students were more influenced by frequent reliance on scientific literature, family and YouTube channels as information sources, whereas female students were more influenced by confidence in vaccine safety, social influence of colleagues and media, and financial status. These results indicate the need to develop tailored communication strategies that respond to the distinct information needs and social contexts of male and female students, and promote the use of reliable information sources. Our results contribute to the growing evidence on sex-specific determinants of HPV vaccine uptake across different settings and cultural contexts. Recognizing and addressing these differences can improve HPV vaccination coverage, reduce the burden of HPV-related diseases and enhance the effectiveness of preventive programs targeted at young adults.

## Figures and Tables

**Table 1:** Sociodemographic characteristics of study participants by sex.

	All Participants	Male	Female	*p* Value
	*N* = 1952	*N* = 423	*N* = 1529	
Age (years)	21.8 (18.0–27.0)	21.7 (18.0–27.0)	21.9 (18.0–27.0)	0.200
Faculty group *:				0.047
Technology and engineering sciences	422 (21.6%)	146 (34.5%)	276 (18.1%)	
Sciences and mathematics	176 (9.0%)	41 (9.7%)	135 (8.8%)	
Medical sciences	484 (24.8%)	73 (17.3%)	411 (26.9%)	
Social sciences and humanities	759 (38.9%)	138 (32.6%)	621 (40.6%)	
Arts	33 (1.7%)	3 (0.7%)	30 (2.0%)	
Self-reported religiousness:				
Completely nonreligious	333 (19.9%)	99 (25.9%)	234 (18.1%)	<0.01
Moderately nonreligious	152 (9.1%)	41 (10.7%)	111 (8.6%)	0.224
Neither nonreligious nor religious	271 (16.2%)	47 (12.3%)	224 (17.4%)	<0.05
Moderately religious	489 (29.2%)	104 (27.2%)	385 (29.8%)	0.338
Completely religious	428 (25.6%)	91 (23.8%)	337 (26.1%)	0.386
Self-assessed financial status:				<0.05
Very good	163 (8.4%)	29 (6.9%)	134 (8.8%)	
Good	673 (34.5%)	167 (39.5%)	506 (33.1%)	
Average	986 (50.5%)	199 (47.0%)	787 (51.5%)	
Bad	85 (4.4%)	16 (3.8%)	69 (4.5%)	
Very bad	7 (0.4%)	4 (0.9%)	3 (0.2%)	
I would rather not say	38 (1.9%)	8 (1.9%)	30 (2.0%)	
Relationship status:				<0.01
Single (including divorced)	1112 (58.8%)	267 (65.9%)	845 (56.9%)	
In a long-term relationship (including married)	727 (38.4%)	123 (30.4%)	604 (40.6%)	
Other–It’s complicated	52 (2.7%)	15 (13.7%)	37 (2.5%)	
HPV vaccination status				<0.001
Vaccinated	1062 (54.4%)	164 (38.8%)	898 (58.7%)	
Non-vaccinated	890 (45.6%)	259 (61.2%)	631 (41.3%)	

* Classification of faculties is based on the official organization of faculties at the University of Belgrade.

**Table 2:** Beliefs about HPV vaccine efficacy by sex and vaccination status.

	All Participants *N* = 1952	Male				Female				*p* Value (Male vs. Female)
		Vaccinated *N* = 164	Not Vaccinated *N* = 259	Total *N* = 423	*p* Value (Vaccinated vs. Non-Vaccinated)	Vaccinated *N* = 898	Not Vaccinated *N* = 631	Total *N* = 1529	*p* Value (Vaccinated vs. Non-Vaccinated)	
I believe that HPV vaccine is important for my health.					<0.001				<0.001	<0.001
Strongly disagree	42 (2.2%)	3 1.8%	17 6.6%	20 4.7%		00.0%	22 3.5%	22 1.4%		
Disagree	47 (2.4%)	4 2.4%	16 6.2%	20 4.7%		1 0.1%	26 4.1%	27 1.8%		
Neither disagree nor agree	291 (14.9%)	15 9.1%	93 35.9%	108 25.5%		16 1.8%	167 26.5%	183 12.0%		
Agree	396 (20.3%)	30 18.3%	72 27.8%	102 24.1%		113 12.6%	181 28.7%	294 19.2%		
Strongly agree	1176 (60.2%)	112 68.3%	61 23.6%	173 40.9%		768 85.5%	235 37.2%	1003 65.6%		
I believe that HPV vaccine can prevent HPV infection.					<0.001				<0.001	<0.01
Strongly disagree	35 (1.8%)	4 2.4%	11 4.2%	15 3.5%		9 1.0%	11 1.7%	20 1.3%		
Disagree	43 (2.2%)	3 1.8%	7 2.7%	10 2.4%		11 1.2%	22 3.5%	33 2.2%		
Neither disagree nor agree	231 (11.8%)	8 4.9%	51 19.7%	59 13.9%		37 4.1%	135 21.4%	172 11.2%		
Agree	571 (29.3%)	29 17.7%	80 30.9%	109 25.8%		242 26.9%	220 34.9%	462 30.2%		
Strongly agree	1072 (54.9%)	120 73.2%	110 42.5%	230 54.4%		599 66.7%	243 38.5%	842 55.1%		
I believe that HPV vaccine protects from oncogenic (those that cause cancer) strains of HPV viruses.					<0.001				<0.001	<0.001
Strongly disagree	26 (1.3%)	3 1.8%	7 2.7%	10 2.4%		1 0.1%	15 2.4%	16 1.0%		
Disagree	32 (1.6%)	1 0.6%	11 4.2%	12 2.8%		3 0.3%	17 2.7%	20 1.3%		
Neither disagree nor agree	297 (15.2%)	17 10.4%	87 33.6%	104 24.6%		32 3.6%	161 25.5%	193 12.6%		
Agree	461 (23.6%)	27 16.5%	63 24.3%	90 21.3%		170 18.9%	201 31.9%	371 24.3%		
Strongly agree	1136 (58.2%)	116 70.7%	91 35.1%	207 48.9%		692 77.1%	237 37.6%	929 60.8%		
Perceived HPV vaccine efficacy (total score)	4.34 (SD = 0.81)	4.53 (SD = 0.77)	3.81 (SD = 0.94)	4.09 (SD = 0.94)		4.71 (SD = 0.45)	3.99 (SD = 0.88)	4.41 (SD = 0.75)		<0.001
		Total score—Vaccinated vs. non-vaccinated males: *p* < 0.001				Total score—Vaccinated vs. non-vaccinated females: *p* < 0.001				

**Table 3:** Beliefs about HPV vaccine safety by sex and vaccination status.

	All Participants *N* = 1952	Male				Female				*p* Value (Male vs. Female)
		Vaccinated *N* = 164	Not Vaccinated *N* = 259	Total *N* = 423	*p* Value (Vaccinated vs. Non-Vaccinated)	Vaccinated *N* = 898	Not Vaccinated *N* = 631	Total *N* = 1529	*p* Value (Vaccinated vs. Non-Vaccinated)	
Overall, I believe that vaccines are safe.					<0.001				<0.001	<0.01
Strongly disagree	39 (2.0%)	3 1.8%	15 5.8%	18 4.3%		2 0.2%	19 3.0%	21 1.4%		
Disagree	77 (3.9%)	7 4.3%	14 5.4%	21 5.0%		10 1.1%	46 7.3%	56 3.7%		
Neither disagree nor agree	340 (17.4%)	15 9.1%	60 23.2%	75 17.7%		88 9.8%	177 28.1%	265 17.3%		
Agree	606 (31.0%)	45 27.4%	91 35.1%	136 32.2%		273 30.4%	197 31.2%	470 30.7%		
Strongly agree	890 (45.6%)	94 57.3%	79 30.5%	173 40.9%		525 58.5%	192 30.4%	717 46.9%		
I am concerned that HPV vaccine might cause infertility.					<0.001				<0.001	<0.001
Strongly disagree	898 (46.0%)	84 51.2%	82 31.7%	166 39.2%		572 63.7%	160 25.4%	732 47.9%		
Disagree	386 (19.8%)	30 18.3%	48 18.5%	78 18.4%		190 21.2%	118 18.7%	308 20.1%		
Neither disagree nor agree	417 (21.4%)	26 15.9%	98 37.8%	124 29.3%		86 9.6%	207 32.8%	293 19.2%		
Agree	152 (7.8%)	14 8.5%	17 6.6%	31 7.3%		25 2.8%	96 15.2%	121 7.9%		
Strongly agree	99 (5.1%)	10 6.1%	14 5.4%	24 5.7%		25 2.8%	50 7.9%	75 4.9%		
I believe that HPV vaccine is safe.										<0.001
Strongly disagree	39 (2.0%)	3 1.8%	13 5.0%	16 3.8%	<0.001	3 0.3%	20 3.2%	23 1.5%	<0.001	
Disagree	59 (3.0%)	5 3.0%	21 8.1%	26 6.1%		3 0.3%	30 4.8%	33 2.2%		
Neither disagree nor agree	337 (17.3%)	11 6.7%	74 28.6%	85 20.1%		30 3.3%	222 35.2%	252 16.5%		
Agree	505 (25.9%)	41 25.0%	74 28.6%	115 27.2%		223 24.8%	167 26.5%	390 25.5%		
Strongly agree	1012 (51.8%)	104 63.4%	77 29.7%	181 42.8%		639 71.2%	192 30.4%	831 54.3%		
Serious adverse reactions following HPV vaccination are extremely rare.					<0.001				<0.001	<0.001
Strongly disagree	35 (1.8%)	1 0.6%	8 3.1%	9 2.1%		15 1.7%	11 1.7%	26 1.7%		
Disagree	76 (3.9%)	4 2.4%	14 5.4%	18 4.3%		24 2.7%	34 5.4%	58 3.8%		
Neither disagree nor agree	636 (32.6%)	36 22.0%	142 54.8%	178 42.1%		134 14.9%	324 51.3%	458 30.0%		
Agree	500 (25.6%)	45 27.4%	52 20.1%	97 22.9%		265 29.5%	138 21.9%	403 26.4%		
Strongly agree	705 (36.1%)	78 47.6%	43 16.6%	121 28.6%		460 51.2%	124 19.7%	584 38.2%		
HPV vaccines have not been sufficiently studied for us to be certain about their safety.					<0.001				<0.001	<0.001
Strongly disagree	791 (40.5%)	78 47.6%	61 23.6%	139 32.9%		521 58.0%	131 20.8%	652 42.6%		
Disagree	448 (23.0%)	35 21.3%	44 17.0%	79 18.7%		237 26.4%	132 20.9%	369 24.1%		
Neither disagree nor agree	465 (23.8%)	23 14.0%	117 45.2%	140 33.1%		97 10.8%	228 36.1%	325 21.3%		
Agree	156 (8.0%)	12 7.3%	25 9.7%	37 8.7%		26 2.9%	93 14.7%	119 7.8%		
Strongly agree	92 (4.7%)	16 9.8%	12 4.6%	28 6.6%		17 1.9%	47 7.4%	64 4.2%		
Perceived HPV vaccine safety (total score)	4.01 (SD = 0.83)	0.417 (SD = 0.81)	3.61 (SD = 0.84)	3.82 (SD = 0.87)		4.43 (SD = 0.57)	3.56 (SD = 0.84)	4.06 (SD = 0.81)		<0.001
		Total score—Vaccinated vs. non-vaccinated males: *p* < 0.001				Total score—Vaccinated vs. non-vaccinated females: *p* < 0.001				

**Table 4:** Beliefs about the danger of HPV vaccine-preventable diseases by sex and vaccination status.

	All Participants *N* = 1952	Male				Female				*p* Value (Male vs. Female)
		Vaccinated *N* = 164	Not Vaccinated *N* = 259	Total *N* = 423	*p* Value (Vaccinated vs. Non-Vaccinated)	Vaccinated *N* = 898	Not Vaccinated *N* = 631	Total *N* = 1529	*p* Value (Vaccinated vs. Non-Vaccinated)	
I believe that HPV infection is not so common, so I consider myself as being at low risk of contracting it.					<0.001				<0.001	<0.001
Strongly disagree	959 (49.1%)	68 41.5%	60 23.2%	128 30.3%		600 66.8%	231 36.6%	831 54.3%		
Disagree	384 (19.7%)	32 19.5%	42 16.2%	74 17.5%		173 19.3%	137 21.7%	310 20.3%		
Neither disagree nor agree	430 (22.0%)	38 23.2%	114 44.0%	152 35.9%		78 8.7%	200 31.7%	278 18.2%		
Agree	107 (5.5%)	11 6.7%	34 13.1%	45 10.6%		20 2.2%	42 6.7%	62 4.1%		
Strongly agree	72 (3.7%)	15 9.1%	9 3.5%	24 5.7%		27 3.0%	21 3.3%	48 3.1%		
I believe HPV infection can be prevented by other means (etc. by condom).					<0.01				<0.001	<0.001
Strongly disagree	478 (24.5%)	34 20.7%	30 11.6%	64 15.1%		288 32.1%	126 20.0%	414 27.1%		
Disagree	458 (23.5%)	38 23.2%	36 13.9%	74 17.5%		256 28.5%	128 20.3%	384 25.1%		
Neither disagree nor agree	579 (29.7%)	42 25.6%	87 33.6%	129 30.5%		219 24.4%	231 36.6%	450 29.4%		
Agree	326 (16.7%)	35 21.3%	79 30.5%	114 27.0%		103 11.5%	109 17.3%	212 13.9%		
Strongly agree	111 (5.7%)	15 9.1%	27 10.4%	42 9.9%		32 3.6%	37 5.9%	69 4.5%		
Diseases caused by HPV (cancer of the cervix, vagina, vulva, anus, penis, oral cavity, and genital warts) are not that common.										<0.001
Strongly disagree	961 (49.2%)	68 41.5%	54 20.8%	122 28.8%	<0.001	583 64.9%	256 40.6%	839 54.9%	<0.001	
Disagree	470 (24.1%)	43 26.2%	62 23.9%	105 24.8%		202 22.5%	163 25.8%	365 23.9%		
Neither disagree nor agree	381 (19.5%)	32 19.5%	110 42.5%	142 33.6%		75 8.4%	164 26.0%	239 15.6%		
Agree	72 (3.7%)	10 6.1%	27 10.4%	37 8.7%		9 1.0%	26 4.1%	35 2.3%		
Strongly agree	68 (3.5%)	11 6.7%	6 2.3%	17 4.0%		29 3.2%	22 3.5%	51 3.3%		
HPV infection is not dangerous because it usually resolves spontaneously.					<0.001				<0.001	<0.001
Strongly disagree	1004 (51.4%)	68 41.5%	74 28.6%	142 33.6%		580 64.6%	282 44.7%	862 56.4%		
Disagree	436 (22.3%)	37 22.6%	68 26.3%	105 24.8%		189 21.0%	142 22.5%	331 21.6%		
Neither disagree nor agree	396 (20.3%)	41 25.0%	101 39.0%	142 33.6%		88 9.8%	166 26.3%	254 16.6%		
Agree	62 (3.2%)	6 3.7%	13 5.0%	19 4.5%		19 2.1%	24 3.8%	43 2.8%		
Strongly agree	54 (2.8%)	12 7.3%	3 1.2%	15 3.5%		22 2.4%	17 2.7%	39 2.6%		
Perceived danger of the HPV vaccine-preventable diseases (total score)	3.94 (SD = 0.86)	3.70 (SD = 1.04)	3.39 (SD = 0.78)	3.51 (SD = 0.90)		4.27 (SD = 0.72)	3.78 (SD = 0.85)	4.06 (SD = 0.81)		<0.001
		Total score—Vaccinated vs. non-vaccinated males: *p* < 0.001				Total score—Vaccinated vs. non-vaccinated females: *p* < 0.001				

**Table 5:** Frequency of use of information sources about vaccines by sex and vaccination status.

	All Participants *N* = 1952	Male				Female				*p* Value (Male vs. Female)
		Vaccinated *N* = 164	Not Vaccinated *N* = 259	Total *N* = 423	*p* Value (Vaccinated vs. Non-Vaccinated)	Vaccinated *N* = 898	Not Vaccinated *N* = 631	Total *N* = 1529	*p* Value (Vaccinated vs. Non-Vaccinated)	
Scientific literature:					<0.001				<0.001	<0.001
Never	529 (27.1%)	40 24.4%	108 41.7%	148 35.0%		190 21.2%	191 30.3%	381 24.9%		
Rarely	383 (19.6%)	30 18.3%	54 20.8%	84 19.9%		172 19.2%	127 20.1%	299 19.6%		
Sometimes	593 (30.4%)	41 25.0%	67 25.9%	108 25.5%		281 31.3%	204 32.3%	485 31.7%		
Often	292 (15.0%)	28 17.1%	14 5.4%	42 9.9%		168 18.7%	82 13.0%	250 16.4%		
Regularly	155 (7.9%)	25 15.2%	16 6.2%	41 9.7%		87 9.7%	27 4.3%	114 7.5%		
National TV channels:					<0.01				0.730	<0.001
Never	668 (34.2%)	72 43.9%	119 45.9%	191 45.2%		283 31.5%	194 30.7%	477 31.2%		
Rarely	511 (26.2%)	35 21.3%	59 22.8%	94 22.2%		254 28.3%	163 25.8%	417 27.3%		
Sometimes	553 (28.3%)	33 20.1%	72 27.8%	105 24.8%		256 28.5%	192 30.4%	448 29.3%		
Often	165 (8.5%)	15 9.1%	6 2.3%	21 5.0%		82 9.1%	62 9.8%	144 9.4%		
Regularly	55 (2.8%)	9 5.5%	3 1.2%	12 2.8%		23 2.6%	20 3.2%	43 2.8%		
Regional TV channels:					0.241				0.270	<0.001
Never	802 (41.1%)	85 51.8%	136 52.5%	221 52.2%		353 39.3%	228 36.1%	581 38.0%		
Rarely	484 (24.8%)	35 21.3%	54 20.8%	89 21.0%		239 26.6%	156 24.7%	395 25.8%		
Sometimes	502 (25.7%)	30 18.3%	59 22.8%	89 21.0%		233 25.9%	180 28.5%	413 27.0%		
Often	115 (5.9%)	6 3.7%	6 2.3%	12 2.8%		55 6.1%	48 7.6%	103 6.7%		
Regularly	49 (2.5%)	8 4.9%	4 1.5%	12 2.8%		18 2.0%	19 3.0%	37 2.4%		
Internet portals:					<0.01				<0.001	<0.001
Never	266 (13.6%)	31 18.9%	68 26.3%	99 23.4%		71 7.9%	96 15.2%	167 10.9%		
Rarely	248 (12.7%)	23 14.0%	57 22.0%	80 18.9%		92 10.2%	76 12.0%	168 11.0%		
Sometimes	649 (33.2%)	48 29.3%	83 32.0%	131 31.0%		295 32.9%	223 35.3%	518 33.9%		
Often	554 (28.4%)	43 26.2%	38 14.7%	81 19.1%		299 33.3%	174 27.6%	473 30.9%		
Regularly	235 (12.0%)	19 11.6%	13 5.0%	32 7.6%		141 15.7%	62 9.8%	203 13.3%		
YouTube channels:					0.701				0.081	<0.01
Never	689 (35.8%)	67 40.9%	118 45.6%	185 43.7%		298 33.2%	215 34.1%	513 33.6%		
Rarely	402 (20.6%)	35 21.3%	50 19.3%	85 20.1%		191 21.3%	126 20.0%	317 20.7%		
Sometimes	509 (26.1%)	35 21.3%	59 22.8%	94 22.2%		225 25.1%	190 30.1%	415 27.1%		
Often	243 (12.4%)	19 11.6%	24 9.3%	43 10.2%		131 14.6%	69 10.9%	200 13.1%		
Regularly	100 (5.1%)	8 4.9%	8 3.1%	16 3.8%		53 5.9%	31 4.9%	84 5.5%		
Social networks (Instagram, Facebook, Viber, Twitter, WhatsApp):					<0.05				<0.001	<0.001
Never	300 (15.4%)	38 23.2%	82 31.7%	120 28.4%		87 9.7%	93 14.7%	180 11.8%		
Rarely	305 (15.6%)	31 18.9%	62 23.9%	93 22.0%		118 13.1%	94 14.9%	212 13.9%		
Sometimes	587 (30.1%)	44 26.8%	69 26.6%	113 26.7%		252 28.1%	222 35.2%	474 31.0%		
Often	483 (24.7%)	33 20.1%	30 11.6%	63 14.9%		269 30.0%	151 23.9%	420 27.5%		
Regularly	277 (14.2%)	18 11.0%	16 6.2%	34 8.0%		172 19.2%	71 11.3%	243 15.9%		
Family:					<0.001				<0.001	<0.001
Never	397 (20.3%)	47 28.7%	104 40.2%	151 35.7%		125 13.9%	121 19.2%	246 16.1%		
Rarely	361 (18.5%)	25 15.2%	58 22.4%	83 19.6%		150 16.7%	128 20.3%	278 18.2%		
Sometimes	617 (31.6%)	41 25.0%	71 27.4%	112		278 31.0%	227 36.0%	505		
Often	358 (18.3%)	26 15.9%	18 6.9%	26.5%		211 23.5%	103 16.3%	33.0%		
Regularly	219 (11.2%)	25 15.2%	8 3.1%	44 10.4%		134 14.9%	52 8.2%	314 20.5%		
Friends:										<0.001
Never	256 13.1%	26 15.9%	87 33.6%	113 26.7%	<0.001	59 6.6%	84 13.3%	143 9.4%	<0.001	
Rarely	321 16.4%	31 18.9%	48 18.5%	79 18.7%		133 14.8%	109 17.3%	242 15.8%		
Sometimes	673 34.5%	53 32.3%	73 28.2%	126 29.8%		305 34.0%	242 38.4%	547 35.8%		
Often	485 24.8%	34 20.7%	37 14.3%	71 16.8%		271 30.2%	143 22.7%	414 27.1%		
Regularly	217 11.1%	20 12.2%	14 5.4%	34 8.0%		130 14.5%	53 8.4%	183 12.0%		
Your chosen physician, or physician you visit most often:					<0.001				<0.001	<0.001
Never	400 20.5%	39 23.8%	95 36.7%	134 31.7%		137 15.3%	129 20.4%	266 17.4%		
Rarely	357 18.3%	28 17.1%	53 20.5%	81 19.1%		139 15.5%	137 21.7%	276 18.1%		
Sometimes	610 31.3%	44 26.8%	80 30.9%	124 29.3%		268 29.8%	218 34.5%	486 31.8%		
Often	353 18.1%	27 16.5%	18 6.9%	45 10.6%		214 23.8%	94 14.9%	308 20.1%		
Regularly	232 11.9%	26 15.9%	13 5.0%	39 9.2%		140 15.6%	53 8.4%	193 12.6%		
Healthcare professionals in media:					<0.01				<0.01	<0.001
Never	431 22.1%	44 26.8%	85 32.8%	129 30.5%		156 17.4%	146 23.1%	302 19.8%		
Rarely	384 19.7%	25 15.2%	51 19.7%	76 18.0%		173 19.3%	135 21.4%	308 20.1%		
Sometimes	645 33.0%	46 28.0%	90 34.7%	136 32.2%		299 33.3%	210 33.3%	509 33.3%		
Often	331 17.0%	29 17.7%	22 8.5%	51 12.1%		178 19.8%	102 16.2%	280 18.3%		
Regularly	161 8.2%	20 12.2%	11 4.2%	31 7.3%		92 10.2%	38 6.0%	130 8.5%		
Religious leaders:					<0.05				<0.001	<0.01
Never	1459 74.7%	125 76.2%	180 69.5%	305 72.1%		731 81.4%	423 67.0%	1154 75.5%		
Rarely	197 10.1%	12 7.3%	26 10.0%	38 9.0%		78 8.7%	81 12.8%	159 10.4%		
Sometimes	242 12.4%	15 9.1%	44 17.0%	59 13.9%		74 8.2%	109 17.3%	183 12.0%		
Often	29 1.5%	4 2.4%	5 1.9%	9 2.1%		9 1.0%	11 1.7%	20 1.3%		
Regularly	25 1.3%	8 4.9%	4 1.5%	12 2.8%		6 0.7%	7 1.1%	13 0.9%		
Government:					0.551				<0.05	0.109
Never	1128 57.8%	90 54.9%	141 54.4%	231 54.6%		553 61.6%	344 54.5%	897 58.7%		
Rarely	375 19.2%	30 18.3%	49 18.9%	79 18.7%		173 19.3%	123 19.5%	296 19.4%		
Sometimes	362 18.5%	32 19.5%	59 22.8%	91 21.5%		138 15.4%	133 21.1%	271 17.7%		
Often	53 2.7%	5 3.0%	5 1.9%	10 2.4%		21 2.3%	22 3.5%	43 2.8%		
Regularly	34 1.7%	7 4.3%	5 1.9%	12 2.8%		13 1.4%	9 1.4%	22 1.4%		

**Table 6:** Perceived lack of information by sex and vaccination status.

	All Participants *N* = 1952	Male				Female				*p* Value (Male vs. Female)
		Vaccinated *N* = 164	Not Vaccinated *N* = 259	Total *N* = 423	*p* Value (Vaccinated vs. Non-Vaccinated)	Vaccinated *N* = 898	Not Vaccinated *N* = 631	Total *N* = 1529	*p* Value (Vaccinated vs. Non-Vaccinated)	
It is hard to make the decision whether to vaccinate against HPV, since there is a lack of information about vaccines.					<0.001				<0.001	<0.05
Strongly disagree	533 27.3%	63 38.4%	58 22.4%	121 28.6%		336 37.4%	76 12.0%	412 26.9%		
Disagree	368 18.9%	35 21.3%	32 12.4%	67 15.8%		215 23.9%	86 13.6%	301 19.7%		
Neither disagree nor agree	598 30.6%	41 25.0%	112 43.2%	153 36.2%		186 20.7%	259 41.0%	445 29.1%		
Agree	305 15.6%	15 9.1%	36 13.9%	51 12.1%		128 14.3%	126 20.0%	254 16.6%		
Strongly agree	148 7.6%	10 6.1%	21 8.1%	31 7.3%		33 3.7%	84 13.3%	117 7.7%		
Incomplete information regarding the HPV vaccines I come across make me confused.					<0.001				<0.001	<0.05
Strongly disagree	574 29.4%	66 40.2%	52 20.1%	118 27.9%		373 41.5%	83 13.2%	456 29.8%		
Disagree	379 19.4%	27 16.5%	37 14.3%	64 15.1%		223 24.8%	92 14.6%	315 20.6%		
Neither disagree nor agree	589 30.2%	40 24.4%	107 41.3%	147 34.8%		196 21.8%	246 39.0%	442 28.9%		
Agree	279 14.3%	23 14.0%	47 18.1%	70 16.5%		80 8.9%	129 20.4%	209 13.7%		
Strongly agree	131 6.7%	8 4.9%	16 6.2%	24 5.7%		26 2.9%	81 12.8%	107 7.0%		
Contradictory information regarding the HPV vaccines I come across make me confused.										<0.01
Strongly disagree	590 30.2%	68 41.5%	63 24.3%	131 31.0%	<0.001	367 40.9%	92 14.6%	459 30.0%	<0.001	
Disagree	360 18.4%	29 17.7%	33 12.7%	62 14.7%		206 22.9%	92 14.6%	298 19.5%		
Neither disagree nor agree	623 31.9%	42 25.6%	120 46.3%	162 38.3%		204 22.7%	257 40.7%	461 30.2%		
Agree	243 12.4%	19 11.6%	28 10.8%	47 11.1%		88 9.8%	108 17.1%	196 12.8%		
Strongly agree	136 7.0%	6 3.7%	15 5.8%	21 5.0%		33 3.7%	82 13.0%	115 7.5%		
I have all the information I need regarding HPV vaccination.					<0.001				<0.001	<0.01
Strongly disagree	173 8.9%	3 1.8%	42 16.2%	45 10.6%		30 3.3%	98 15.5%	128 8.4%		
Disagree	216 11.1%	20 12.2%	38 14.7%	58 13.7%		57 6.3%	101 16.0%	158 10.3%		
Neither disagree nor agree	564 28.9%	37 22.6%	100 38.6%	137 32.4%		172 19.2%	255 40.4%	427 27.9%		
Agree	558 28.6%	49 29.9%	48 18.5%	97 22.9%		346 38.5%	115 18.2%	461 30.2%		
Strongly agree	441 22.6%	55 33.5%	31 12.0%	86 20.3%		293 32.6%	62 9.8%	355 23.2%		
Perceived lack of information (total score)	2.5 (SD = 1.06)	2.22 (SD = 1.01)	2.79 (SD = 0.94)	2.6 (SD = 1.01)		2.13 (SD = 0.96)	3.06 (SD = 0.99)	2.5 (SD = 1.08)		0.193
		Total score—Vaccinated vs. non-vaccinated males: *p* < 0.001				Total score—Vaccinated vs. non-vaccinated females: *p* < 0.001				

**Table 7:** Self-ranked influence of social agents on vaccination intention by sex.

	All Participants *N* = 1952	Male				Female				*p* Value (Male vs. Female)
		Vaccinated *N* = 164	Not Vaccinated *N* = 259	Total *N* = 423	*p* Value (Vaccinated vs. Non-Vaccinated)	Vaccinated *N* = 898	Not Vaccinated *N* = 631	Total *N* = 1529	*p* Value (Vaccinated vs. Non-Vaccinated)	
Own attitudes	1767 90.5%	145 88.4%	226 87.3%	371 87.7%	0.724	832 92.7%	564 89.4%	1396 91.3%	<0.05	<0.05
Family	857 43.9%	69 42.1%	85 32.8%	154 36.4%	0.054	427 47.6%	276 43.7%	703 46.0%	0.141	<0.001
Friends	503 25.8%	44 26.8%	59 22.8%%	103 24.3%	0.344	257 28.6%	143 22.7%	400 26.2%	<0.01	0.451
Peers	74 3.8%	14 8.5%	10 3.9%	24 5.7%	0.043	29 3.2%	21 3.3%	50 3.3%	0.915	<0.05
Faculty colleagues	231 11.8%	25 15.2%	18 6.9%	43 10.2%	<0.01	141 15.7%	47 7.4%	188 12.3%	<0.001	0.230
Community members	14 0.7%	2 1.2%	2 0.8%	4 0.9%	0.643	4 0.4%	6 1.0%	10 0.7%	0.334	0.519
National health authorities (Ministry of health, Institute of Public Health)	255 13.1%	27 16.5%	45 17.4%	72 17.0%	0.808	98 10.9%	85 13.5%	183 12.0%	0.129	<0.01
Religious leaders	17 0.9%	1 0.6%	6 2.3%	7 1.7%	0.256	1 0.1%	9 1.4%	10 0.7%	<0.01	0.070
Healthcare workers	616 31.6%	37 22.6%	75 29.0%	504 33.0%	0.146	297 33.1%	207 32.8%	504 33.0%	0.912	<0.05
Government	7 0.4%	00.0%	6 2.3%	6 1.4%	N/A	1 0.1%	00.0%	1 0.1%	N/A	<0.01
Media (TV, radio, newspapers, internet)	160 8.2%	21 12.8%	19 7.3%	40 9.5%	0.061	90 10.0%	30 4.8%	120 7.8%	<0.001	0.286

**Table 8:** Hierarchical multiple logistic regression analysis of variables significantly associated with vaccination status in the total sample.

Variable	Model 1	Model 2	Model 3	Model 4
	OR (95% CI)	OR (95% CI)	OR (95% CI)	OR (95% CI)
Age	1.11 (1.05–1.18) ***	1.04 (0.98–1.11)	1.05 (0.98–1.12)	1.05 (0.98–1.12)
Religiosity				
Completely nonreligious	2.69 (1.97–3.68) ***	1.29 (0.89–1.85)	1.28 (0.87–1.88)	1.27 (0.86–1.88)
Moderately nonreligious	2.08 (1.40–3.07) ***	1.15 (0.74–1.80)	1.19 (0.74–1.90)	1.16 (0.72–1.87)
Neither nonreligious nor religious	1.93 (1.39–2.66) ***	1.50 (1.03–2.19) *	1.46 (0.99–2.14)	1.46 (0.99–2.15)
Moderately religious	1.62 (1.23–2.13) **	1.36 (0.99–1.87)	1.39 (0.99–1.92)	1.39 (0.99–1.94)
Completely religious	Ref.	Ref.	Ref.	Ref.
Faculty group:				
Technology and engineering sciences	0.45 (0.37–0.61) ***	0.89 (0.63–1.26)	0.99 (0.69–1.43)	1.03 (0.70–1.50)
Sciences and mathematics	0.47 (0.32–0.70) ***	0.77 (0.50–1.19)	0.88 (0.56–1.39)	0.88 (0.55–1.39)
Medical sciences	Ref.	Ref.	Ref.	Ref.
Social sciences and humanities	0.53 (0.41–0.69) ***	0.87 (0.65–1.18)	0.95 (0.68–1.31)	0.98 (0.70–1.38)
Self-assessed financial status				
Very good	1.99 (0.90–4.43)	1.15 (0.45–2.90)	0.87 (0.34–2.18)	0.77 (0.31–1.95)
Good	2.05 (0.99–4.26)	1.22 (0.52–2.83)	0.98 (0.42–2.27)	0.84 (0.36–1.94)
Average	1.73 (0.84–3.57)	1.17 (0.51–2.70)	1.01 (0.44–2.31)	0.87 (0.38–1.99)
Poor	0.92 (0.39–2.15)	0.43 (0.16–1.13)	0.41 (0.16–1.08)	0.37 (0.14–1.00)
Very poor	Ref.	Ref.	Ref.	Ref.
Beliefs about vaccine efficacy		2.26 (1.80–2.85) ***	1.97 (1.56–2.49) ***	1.98 (1.56–2.52) ***
Beliefs about vaccine safety		2.36 (1.89–2.95) ***	1.83 (1.42–2.35) ***	1.80 (1.40–2.32) ***
Beliefs about danger of diseases		1.18 (1.01–1.38) *	1.10 (0.93–1.30)	1.09 (0.92–1.29)
Perceived lack of information			0.74 (0.64–0.85) ***	0.73 (0.63–0.85) ***
Scientific literature (frequency of use as a source)			1.09 (0.97–1.23)	1.11 (0.99–1.26)
YouTube channels			0.89 (0.79–1.01)	0.88 (0.77–0.99) *
Social networks (frequency of use as a source)			1.12 (0.99–1.26)	1.12 (0.99–1.26)
Family (frequency of use as a source)			1.20 (1.07–1.35) **	1.11 (0.98–1.26)
Chosen physician (frequency of use as a source)			1.12 (1.00–1.26) *	1.18 (1.05–1.32) **
Religious leaders (frequency of use as a source)			0.87 (0.73–1.04)	0.89 (0.75–1.07)
Family (social influence)				1.64 (1.23–2.18) **
Friends (social influence)				1.09 (0.82–1.45)
Colleagues from faculty (social influence)				1.63 (1.09–2.44) *
Religious leaders (social influence)				0.70 (0.14–3.41)
Media (social influence)				1.81 (1.16–2.82) **
Nagelkerke R^2^	0.088	0.356	0.390	0.403

* *p* < 0.05; ** *p* < 0.01; *** *p* < 0.001.

**Table 9:** Hierarchical multiple logistic regression analysis of variables significantly associated with vaccination status in male participants.

Variable	Model 1	Model 2	Model 3	Model 4
	OR (95% CI)	OR (95% CI)	OR (95% CI)	OR (95% CI)
Religiosity				
Completely nonreligious	1.83 (0.99–3.38)	1.10 (0.53–2.26)	1.03 (0.48–2.20)	1.02 (0.47–2.21)
Moderately nonreligious	1.43 (0.65–3.14)	0.92 (0.39–2.20)	0.90 (0.35–2.27)	0.88 (0.34–2.24)
Neither nonreligious nor religious	1.20 (0.56–2.56)	0.92 (0.41–2.09)	0.86 (0.36–2.03)	0.93 (0.39–2.23)
Moderately religious	0.83 (0.44–1.57)	0.76 (0.38–1.52)	0.64 (0.31–1.33)	0.68 (0.33–1.43)
Completely religious	Ref.	Ref.	Ref.	Ref.
Faculty group:				
Technology and engineering sciences	0.50 (0.27–0.92) *	0.90 (0.46–1.77)	1.55 (0.70–3.44)	1.63 (0.72–3.70)
Sciences and mathematics	0.59 (0.26–1.31)	1.16 (0.47–2.87)	2.02 (0.75–0.55)	2.04 (0.74–5.61)
Medical sciences	Ref.	Ref.	Ref.	Ref.
Social sciences and humanities	0.32 (0.17–0.60) ***	0.54 (0.27–1.06)	0.822 (0.37–1.81)	0.88 (0.39–1.99)
Beliefs about vaccine efficacy		2.48 (1.60–3.83) ***	2.21 (1.42–3.44) ***	2.14 (1.37–3.34) *
Beliefs about vaccine safety		1.24 (0.79–1.94)	0.98 (0.58–1.67)	1.01 (0.59–1.73)
Beliefs about danger of diseases		1.04 (0.77–1.39)	1.05 (0.76–1.45)	1.02 (0.73–1.41)
Perceived lack of information			0.74 (0.54–1.01)	0.71 (0.52–0.98) *
Scientific literature (frequency of use as a source)			1.45 (1.12–1.89) **	1.50 (1.15–1.97) **
YouTube channels (frequency of use as a source)			0.72 (0.55–0.94) *	0.70 (0.53–0.92) *
Family (frequency of use as a source)			1.45 (1.15–1.84) **	1.37 (1.07–1.75) *
Family (social influence)				1.83 (1.03–3.24) *
Colleagues from faculty (social influence)				1.58 (0.66–3.79)
Media (social influence)				1.74 (0.77–3.95)
Nagelkerke R^2^	0.074	0.228	0.313	0.331

* *p* < 0.05; ** *p* < 0.01; *** *p* < 0.001.

**Table 10:** Hierarchical multiple logistic regression analysis of variables significantly associated with vaccination status in female participants.

Variable	Model 1	Model 2	Model 3	Model 4
	OR (95% CI)	OR (95% CI)	OR (95% CI)	OR (95% CI)
Age	1.16 (1.08–1.24) ***	1.08 (0.99–1.17)	1.07 (0.99–1.16)	1.07 (0.99–1.16)
Religiosity				
Completely nonreligious	3.74 (2.54–5.52) ***	1.66 (1.06–2.60) *	1.51 (0.95–2.42)	1.51 (0.94–2.42)
Moderately nonreligious	2.47 (1.55–3.93) ***	1.28 (0.74–2.20)	1.19 (0.68–2.11)	1.14 (0.64–2.03)
Neither nonreligious nor religious	2.10 (1.46–3.02) ***	1.65 (1.07–2.54) *	1.56 (1.00–2.42)	1.53 (0.98–2.39)
Moderately religious	1.88 (1.37–2.57) ***	1.57 (1.08–2.28) *	1.57 (1.07–2.30) *	1.54 (1.05–2.27)
Completely religious	Ref.	Ref.	Ref.	Ref.
Faculty group:				
Technology and engineering sciences	0.50 (0.35–0.71) ***	0.97 (0.64–1.47)	0.99 (0.64–1.54)	1.01 (0.64–1.59)
Sciences and mathematics	0.44 (0.28–0.69) ***	0.61 (0.37–1.02)	0.65 (0.38–1.10)	0.64 (0.37–1.09)
Medical sciences	Ref.	Ref.	Ref.	Ref.
Social sciences and humanities	0.60 (0.45–0.81) ***	1.00 (0.71–1.42)	1.06 (0.73–1.55)	1.10 (0.75–1.62)
Self-assessed financial status				
Very good	1.70 (0.66–4.39)	1.23 (0.41–3.74)	0.84 (0.28–2.59)	0.79 (0.26–2.42)
Good	1.67 (0.70–4.00)	1.04 (0.38–2.89)	0.78 (0.28–2.16)	0.69 (0.25–1.91)
Average	1.34 (0.56–3.17)	1.07 (0.39–2.92)	0.83 (0.30–2.26)	0.74 (0.27–2.02)
Poor	0.55 (0.20–1.50)	0.30 (0.09–0.94) *	0.23 (0.07–0.75) *	0.22 (0.07–0.71) *
Very poor	Ref	Ref.	Ref.	Ref.
Very good	1.70 (0.66–4.39)	1.23 (0.41–3.74)		
Beliefs about vaccine efficacy		2.18 (1.64–2.89) ***	1.96 (1.48–2.62) ***	2.01 (1.50–2.69) ***
Beliefs about vaccine safety		3.14 (2.40–4.13) ***	2.35 (1.74–3.18) ***	2.29 (1.69–3.10) ***
Beliefs about danger of diseases		1.11 (0.91–1.36)	1.05 (0.86–1.30)	1.05 (0.85–1.30)
Perceived lack of information			0.71 (0.60–0.84) ***	0.71 (0.60–0.84) ***
Scientific literature (frequency of use as a source)			1.03 (0.90–1.18)	1.05 (0.911–1.20)
Social networks (frequency of use as a source)			1.08 (0.96–1.23)	1.07 (0.94–1.21)
Family (frequency of use as a source)			1.12 (0.98–1.28)	1.06 (0.91–1.23)
Chosen physician (frequency of use as a source)			1.06 (0.93–1.21)	1.12 (0.98–1.28)
Religious leaders (frequency of use as a source)			0.80 (0.65–0.98) *	0.81 (0.66–1.00)
Family (social influence)				1.45 (1.04–2.03) *
Friends (social influence)				1.24 (0.88–1.73)
Colleagues from faculty (social influence)				1.62 (1.01–2.59) *
Media (social influence)				1.92 (1.10–3.37) *
Nagelkerke R^2^	0.112	0.407	0.429	0.440

* *p* < 0.05; *** *p* < 0.001.

## Data Availability

Data available upon reasonable request to the corresponding author.

## Supplementary Materials

The following supporting information can be downloaded at: https://www.mdpi.com/article/10.3390/ejihpe15090189/s1, Table S1: STROBE checklist.
