# Supplementary material for: Mind the Gap: Sex-Specific Drivers of Human Papillomavirus Vaccination Uptake in Serbian University Students
Source: Eur J Investig Health Psychol Educ. 2025 Sep 19;15(9):189. doi: 10.3390/ejihpe15090189 (PMC12468811; doi:10.3390/ejihpe15090189)
Supplement: Supplementary file 1 [file ejihpe-15-00189-s001.zip › ejihpe-3827980-supplementary.pdf]

Table S1. STROBE Checklist

	Item No	Recommendation	Location in Manuscript
**Title and abstract**	1	(*a*) Indicate the study's design with a commonly used term in the title or the abstract	Abstract
		(*b*) Provide in the abstract an informative and balanced summary of what was done and what was found	Abstract
**Introduction**			
Background/rationale	2	Explain the scientific background and rationale for the investigation being reported	Introduction, paragraphs 1-7
Objectives	3	State specific objectives, including any prespecified hypotheses	Introduction, paragraph 7
**Methods**			
Study design	4	Present key elements of study design early in the paper	Methods, *Study design and setting* subsection
Setting	5	Describe the setting, locations, and relevant dates, including periods of recruitment, exposure, follow-up, and data collection	Methods, *Study design and setting* subsection
Participants	6	*Cross-sectional study*—Give the eligibility criteria, and the sources and methods of selection of participants	Methods, *Participants* subsection
Variables	7	Clearly define all outcomes, exposures, predictors, potential confounders, and effect modifiers. Give diagnostic criteria, if applicable	Methods, *Study instrument and variables* subsection
Data sources/ measurement	8*	For each variable of interest, give sources of data and details of methods of assessment (measurement). Describe comparability of assessment methods if there is more than one group	Methods, *Study instrument and variables* subsection
Bias	9	Describe any efforts to address potential sources of bias	Methods, *Bias* subsection
Study size	10	Explain how the study size was arrived at	/
Quantitative variables	11	Explain how quantitative variables were handled in the analyses. If applicable, describe which groupings were chosen and why	Methods, *Statistical analysis* subsection
Statistical methods	12	(*a*) Describe all statistical methods, including those used to control for confounding	Methods, *Statistical analysis* subsection
		(*b*) Describe any methods used to examine subgroups and interactions	Methods, *Statistical analysis* subsection
		(*c*) Explain how missing data were addressed	/
		Cross-sectional study—If applicable, describe analytical methods taking account of sampling strategy	Not applicable, no complex sampling
		(*e*) Describe any sensitivity analyses	/
**Results**			
Participants	13*	(a) Report numbers of individuals at each stage of study—eg numbers potentially eligible, examined for eligibility, confirmed eligible, included in the study, completing follow-up, and analysed	/
		(b) Give reasons for non-participation at	/

		each stage	
		(c) Consider use of a flow diagram	/
Descriptive data	14*	(a) Give characteristics of study participants (eg demographic, clinical, social) and information on exposures and potential confounders	Results, Table 1
		(b) Indicate number of participants with missing data for each variable of interest	/
Outcome data	15*	*Cross-sectional study*—Report numbers of outcome events or summary measures	Results, Table 1
Main results	16	(*a*) Give unadjusted estimates and, if applicable, confounder-adjusted estimates and their precision (eg, 95% confidence interval). Make clear which confounders were adjusted for and why they were included	Tables 2-10
		(*b*) Report category boundaries when continuous variables were categorized	Tables 2-10
		(*c*) If relevant, consider translating estimates of relative risk into absolute risk for a meaningful time period	/
Other analyses	17	Report other analyses done—eg analyses of subgroups and interactions, and sensitivity analyses	Tables 2-7
**Discussion**			
Key results	18	Summarise key results with reference to study objectives	Discussion, 4.1. – paragraphs 1-2<br>4.2. – paragraph 1<br>4.3. – paragraph 1<br>4.4. – paragraphs 1-2
Limitations	19	Discuss limitations of the study, taking into account sources of potential bias or imprecision. Discuss both direction and magnitude of any potential bias	Discussion, *Limitations* subsection
Interpretation	20	Give a cautious overall interpretation of results considering objectives, limitations, multiplicity of analyses, results from similar studies, and other relevant evidence	Discussion, 4.1. – paragraph 2<br>4.2. – paragraph 2<br>4.3. – paragraph 1<br>4.4. – paragraph 2<br>4.5. – paragraphs 1-4
Generalisability	21	Discuss the generalisability (external validity) of the study results	Discussion, *Limitations* subsection
**Other information**			
Funding	22	Give the source of funding and the role of the funders for the present study and, if applicable, for the original study on which the present article is based	Funding statement
